# Combined Targeting of Glioblastoma Stem-Like Cells by Neutralizing RNA-Bio-Drugs for STAT3

**DOI:** 10.3390/cancers12061434

**Published:** 2020-05-31

**Authors:** Carla Lucia Esposito, Silvia Nuzzo, Maria Luigia Ibba, Lucia Ricci-Vitiani, Roberto Pallini, Gerolama Condorelli, Silvia Catuogno, Vittorio de Franciscis

**Affiliations:** 1 Istituto di Endocrinologia ed Oncologia Sperimentale, Consiglio Nazionale delle Ricerche (CNR), 80145 Naples, Italy; s.catuogno@ieos.cnr.it; 2 IRCCS SDN (Istituto di Ricovero e Cura a Carattere Scientifico, SYNLAB istituto di Diagnostica Nucleare), 80143 Naples, Italy; silvia.nuzzo@synlab.it; 3 Department of Molecular Medicine and Medical Biotechnology, “Federico II” University of Naples, 80131 Naples, Italy; m.ibba@studenti.unina.it (M.L.I.); gecondor@unina.it (G.C.); 4 Department of Oncology and Molecular Medicine, Istituto Superiore di Sanità, 00161 Rome, Italy; lriccivitiani@yahoo.it; 5 Institute of Neurosurgery, Fondazione Policlinico Universitario A. Gemelli IRCCS, Università Cattolica del Sacro Cuore, 00168 Rome, Italy; roberto.pallini@unicatt.it; 6 IRCCS Neuromed (Istituto di Ricovero e Cura a Carattere Scientifico Neuromed)—Istituto Neurologico Mediterraneo, 86077 Pozzilli, Italy

**Keywords:** aptamer, cancer stem cells, glioblastoma, STAT3, targeted delivery

## Abstract

An important drawback in the management of glioblastoma (GBM) patients is the frequent relapse upon surgery and therapy. A likely explanation is that conventional therapies poorly affect a small population of stem-like cancer cells (glioblastoma stem cells, GSCs) that remain capable of repopulating the tumour mass. Indeed, the development of therapeutic strategies able to hit GSCs while reducing the tumour burden has become an important challenge to increase a patient’s survival. The signal transducer and activator of transcription-3 (STAT3) has been reported to play a pivotal role in maintaining the tumour initiating capacity of the GSC population. Therefore, in order to impair the renewal and propagation of the PDGFRβ-expressing GSC population, here we took advantage of the aptamer–siRNA chimera (AsiC), named Gint4.T-STAT3, that we previously have shown to efficiently antagonize STAT3 in subcutaneous PDGFRβ-positive GBM xenografts. We demonstrate that the aptamer conjugate is able to effectively and specifically prevent patient-derived GSC function and expansion. Moreover, because of the therapeutic potential of using miR-10b inhibitors and of the broad expression of the Axl receptor in GBM, we used the GL21.T anti-Axl aptamer as the targeting moiety for anti-miR-10b, showing that, in combination with the STAT3 AsiC, the aptamer–miR-10b antagonist treatment further enhances the inhibition of GSC sphere formation. Our results highlight the potential to use a combined approach with targeted RNA therapeutics to inhibit GBM tumour dissemination and relapse.

## 1. Introduction

Glioblastoma (GBM) is the most common primary brain tumour with a very dismal prognosis despite advances in surgical and medical neuro-oncology [1]. GBM is an infiltrating and highly heterogeneous tumour that contains a small population of stem-like cells with an undifferentiated phenotype that retains stemness properties, including the ability to undergo self-renewal by symmetric cell division and differentiate by asymmetric division, repopulating the bulk tumour mass [2,3]. This population is characterized by an enhanced capacity to initiate tumour formation in vivo and resistance to conventional therapies, being considered as mainly responsible for tumour propagation and recurrence [4,5,6,7]. Therefore, the development of therapeutic options able to target the resistant stem-like cells represents an important challenge to generate effective approaches able to render the tumours unable to maintain themselves or grow. Indeed, the deregulated activity and expression of few transcription factors and microRNAs (miRs), including STAT3 and miR-10b, is critical to initiate and sustain the GSC population [8,9].

In the central nervous system, STAT3 is involved in several processes, such as early development and embryonic stem cell biology [10,11]. This factor is activated by various cytokines and growth factors’ signal cascades and, upon tyrosine phosphorylation, moves into the nuclei where it regulates the expression of a wide range of genes involved in the cell cycle, survival, angiogenesis and immune response. STAT3 abnormal activation has been reported to be involved in the progression of several cancer types, including GBM [12,13,14,15,16]. In addition, its inhibition resulted in an effective alteration of GSC sphere formation and stem-like growth potential [17,18,19], and the pathway has been showed to play a crucial role in GSC chemo and radio-resistance [20,21,22]. These studies indicate STAT3 as a highly promising therapeutic target for GBM able to affect both bulk tumour cells and resistant GSCs, enhancing the success of the treatment. 

Further, a critical role in GBM has been demonstrated for miR-10b, which is highly expressed in this tumour and acts as an oncomiR to promote cancer stem cell propagation [9,23,24]. Thus, its inhibition shows a powerful therapeutic potential. 

We and others have recently described the use of aptamer-based RNA molecules able to selectively drive a small interfering RNA (siRNA) against STAT3 to GBM cells [25,26]. Aptamers are short oligonucleotides able to bind with high affinity and specificity to their targets by acquiring a structured folding. They hold great promise as antagonists of cancer-associated proteins as well as delivery carriers of secondary reagents to target cells [27,28]. Indeed, aptamers against cell surface receptors may inhibit the receptor signalling and be internalized into the cell cytoplasm in a receptor-mediated manner. The last function permits their successful application as delivery vehicles of different therapeutic cargoes, including anti-cancer drugs, toxins, and siRNA or miRNA molecules [29,30,31]. This allows the cargos’ action to be restricted to receptor-expressing target tissues with a consequent reduction of unwanted off-target effects.

In our previous report [25], we used a nuclease-resistant internalizing RNA aptamer, named Gint4.T, to bind and inhibit the platelet-derived growth factor β receptor (PDGFRβ) [32], and then designed an AsiC (Gint4.T-STAT3) for the delivery of a STAT3 siRNA to GBM cells, inhibiting tumour cell growth. Given the key role of STAT3 in GSC propagation and the importance in targeting this population for effective anti-cancer therapies, here our primary objective was to address the functional characterization of Gint4.T-STAT3 on GSCs. Indeed, PDGFRβ is frequently overexpressed in GBM and it is preferentially associated with the self-renewing GSCs [33,34]. We thus hypothesized that Gint4.T-STAT3 could have been used to reduce STAT3 levels in GSCs and alter their function. 

We demonstrate that the AsiC efficiently deliver STAT3 siRNA to PDGFRβ-positive patient-derived GSC primary cell lines, hampering cell survival and migration. Further, we explored the therapeutic potential on GSCs of STAT3 and miR-10b combined inhibition. We used as a targeting moiety for a miR-10b antagonist (anti-miR-10b), the GL21.T aptamer, an inhibitor ligand specific for the receptor tyrosine kinase (RTK) Axl [35], which is expressed in several tumours, including GBM, and also implicated in GSC maintenance [36]. We show that the combined treatment of Gint4.T-STAT3 and GL21.T–anti-miR-10b complexes drastically abrogates the propagation of GSCs.

## 2. Results

### 2.1. Functional Delivery of STAT3-siRNA in Primary GSCs

In order to determine whether the Gint4.T-STAT3 chimera (Figure S1a) inhibits the propagation of the stem-like cells population, as a first attempt we evaluated the targeting efficacy in patient-derived primary human GSCs. We selected three well-characterized primary GBM-derived neurospheres and cultured them as previously reported [37,38,39,40,41], named GSC#83, GSC#61, and GSC#1. All the three lines are positive for the PDGFRβ aptamer target (Figure S1b and [24]) and express comparable levels of STAT3 (Figure S1b,c). Even if standard procedures to culture GSCs using adherent conditions have been successfully described [3,42], we used free-floating neurospheres that are considered to be a specific growing characteristic of GSCs [43]. Further, the effects of aptamer/chimera treatments in a 3D cell environment would better reflect the physiological conditions for penetration of the molecules. 

**Figure 1:**
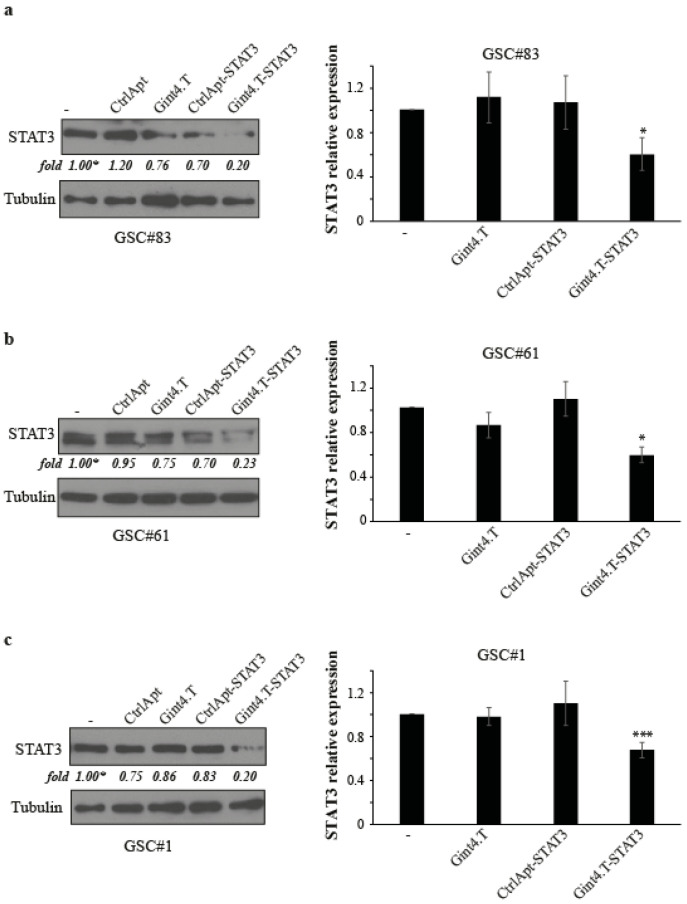
Gint4.T-mediated delivery of STAT3 siRNA in primary GSCs. (**a**–**c**) Primary GSCs (PDGFRβ^+^) were treated with 400 nmol/L Gint4.T, Gint4.T-STAT3, control aptamer (CtrlApt), or control chimera (CtrlApt-STAT3) as indicated. After 72 h, the STAT3 protein (left panels) or mRNA (right panels) levels were analysed by immunoblotting or RT-qPCR, respectively. Anti-tubulin antibody was used as an immunoblot loading control. Values below the blots indicate quantization relative to the untreated (“−”) sample, labelled with an asterisk normalized on the loading control signals. Error bars depict the mean ± SD on two experimental replicates. Statistics were calculated using one-way ANOVA: *, *p* < 0.05; ***, *p* < 0.001, (Gint4.T-STAT3 versus control chimera). Whole blots of Figure 1 are shown in Figure S6.

Upon dissociation, we treated GSCs with Gint4.T-STAT3 AsiC (at 400 nmol/L) and the levels of STAT3 protein and mRNA were determined following 72 h by immunoblotting and quantitative reverse transcription polymerase chain reaction (RT-qPCR), respectively. As shown in Figure 1, the conjugate treatment resulted in an efficient reduction of STAT3 both at the protein (about 70%) and mRNA (about 40%) levels as compared to cells treated with a control conjugate containing a scrambled unrelated aptamer linked to siSTAT3 (CtrlApt-STAT3) in all three lines analysed. These results indicate that the AsiC effectively delivers a functional STAT3 siRNA into PDGFRβ positive GSCs. 

### 2.2. Gint4.T-STAT3 as Inhibitor of Primary GSC Propagation

**Figure 2:**
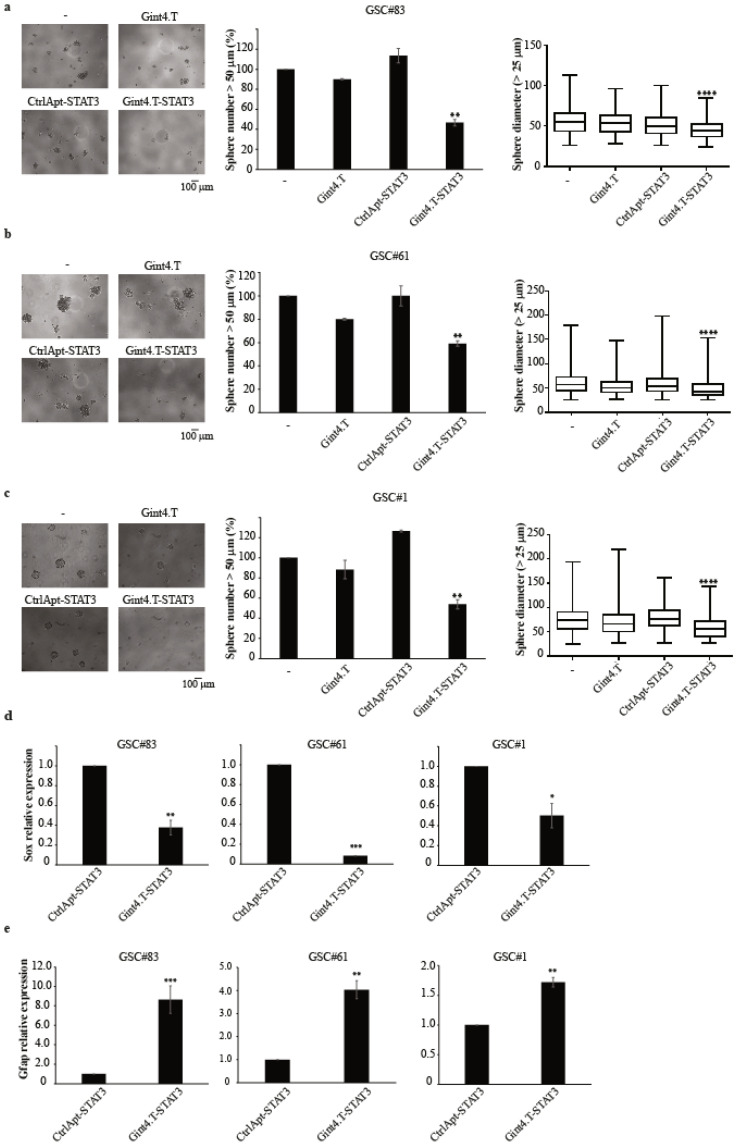
The effect of Gint4.T-STAT3 on primary GSC tumour sphere formation and stemness. (**a**–**c**) Sphere formation of indicated primary GSC-derived tumour spheres (PDGFRβ^+^) left untreated (−) or treated with Gint4.T, Gint4.T-STAT3, or CtrlApt-STAT3. Left panels are representative micrographs; middle panels are spheres with a diameter > 50 μm and were counted and expressed as percentage relative to the untreated samples (−), set to 100%. Vertical bars depict the mean ± SD; and the right panels are boxplot representations of the diameter measures (spheres with a diameter > 25 μm). Statistics of the conjugate treatment versus the control sample using one-way ANOVA: **, *p* < 0.01; ***, *p* < 0.001 ****; *p* < 0.0001. (**d**) Levels of Sox were measured by RT-qPCR (left) or immunoblot (right) in primary GSC-derived tumour spheres (PDGFRβ^+^) treated for 10 days with Gint4.T-STAT3 or control conjugates. Values below the blots indicate quantization relative to the controls, labelled with an asterisk normalized on anti-vinculin signals as a loading control. (**e**) GFAP levels by RT-qPCR after 10 days of GSC treatment with Gint4.T-STAT3 or control conjugates. In (**d**,**e**), statistics for the conjugate treatment versus the control sample were obtained by Student’s *t*-tests: *, *p* < 0.05; **; *p* < 0.01; ***; *p* < 0.001. Vertical bars depict the mean ± SD on replicates (*n* = 2). Whole blots of Figure 2d are shown in Figure S7.

Next, we determined whether Gint4.T-STAT3 could antagonize stem-like GBM cell propagation. The three GSCs were dissociated, treated with the conjugate, and left to form clonal spheres for ten days. We found that the AsiC treatment effectively inhibits tumour sphere formation, reducing the number of spheres (>50 μm diameter) to approximately 50% (Figure 2a–c). In addition, the median size of the spheres (>25 μm diameter) was significantly reduced upon conjugate treatment (Figure 2a–c). Notably, the treatments with the control conjugate did not affect sphere formation, thus indicating that the functional effects depend on Gint4.T-mediated delivery of STAT3 siRNA. 

In addition, we found that the inhibition of the self-renewal potential of the AsiC-treated spheres correlates with the reduction of the stem-cell associated gene SRY-Box 2 (Sox-2), as detected by RT-qPCR and immunoblot (Figure 2d). Conversely, a clear increase in the differentiation marker Glial fibrillary acidic protein (GFAP) levels (Figure 2e) was detected upon Gint4.T-STAT3 treatment in all the three GSCs tested. 

**Figure 3:**
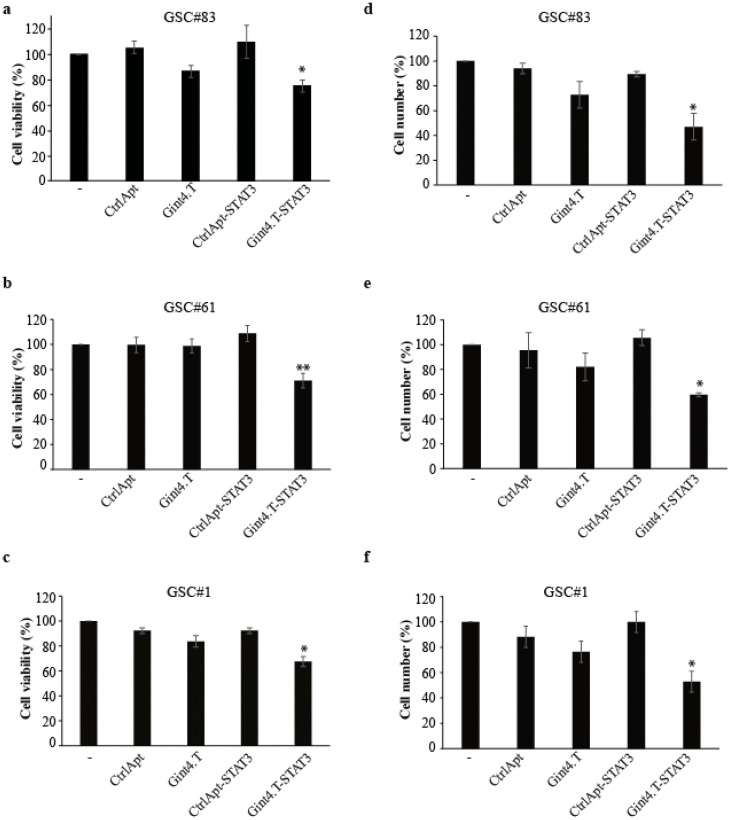
Gint4.T-STAT3 effect on GSC growth. (**a**–**f**) Indicated GSCs (PDGFRβ^+^) were left untreated (−) or treated with indicated aptamer or conjugates (400 nmol/L) for 72 h. (**a**–**c**) Cell viability was measured and expressed as the percentage of the viable cells with respect to the untreated cells. (**d**–**f**) The cell number was counted and expressed as the percentage relative to the untreated cells. In (**a**–**f**), statistics were obtained by one-way ANOVA: *, *p* < 0.05; **, *p* < 0.01 (Gint4.T-STAT3 versus control sample). Vertical bars depict the mean ± SD (*n* = 2).

To further investigate the Gint4.T-STAT3 functional effect, we measured the cell viability and cell count of treated GSCs. In all the three lines, the MTT analysis showed that the AsiC treatment induces a 20–30% reduction of cell viability (Figure 3a–c). In addition, the cell count reached about 60–70% compared to the untreated cells or control conjugate upon Gint4.T-STAT3 treatment (Figure 3d–f). 

Taken together, the results show the AsiC treatment effectively inhibits tumour sphere formation, reducing the cell number and viability.

### 2.3. Gint4.T-STAT3 as Inhibitor of Primary GSC Migration/Invasion

**Figure 4:**
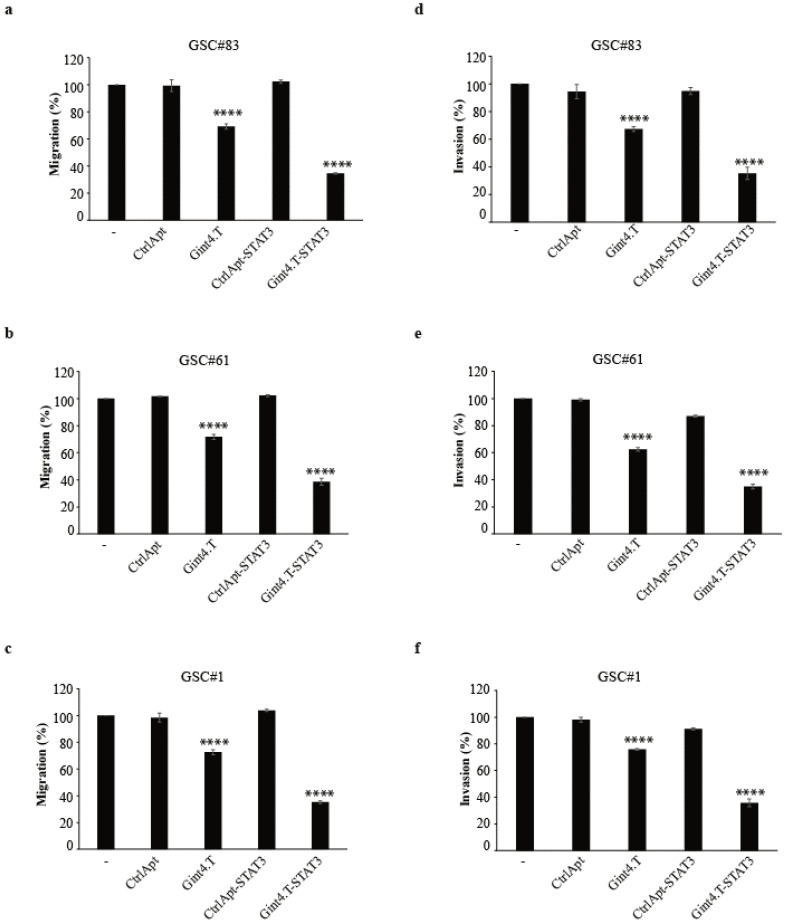
Gint4.T-STAT3 effect on GSC migration and invasion. Cell motility (**a**–**c**) or invasion (**d**–**f**) of indicated GSCs (PDGFRβ^+^) left untreated or treated with indicated aptamers or conjugates (400 nmol/L) for 24 h was analysed. The results are expressed as the percentage of the migrated/invaded cells with respect to the untreated cells. In (**a**–**f**), vertical bars indicate the standard deviation values (*n* = 3). Statistics were obtained by one-way ANOVA (versus control conjugate): ****, *p* < 0.0001.

Stem-like cancer cells are endowed with a high motility potential and can migrate in vitro in the presence of a chemoattractant stimulus. Since we have previously reported that the Gint4.T aptamer hampers cell migration [32] and that the aptamer synergizes with STAT3 siRNA to interfere with cell migration of differentiated GBM cells [25], we then determined whether this function might be as well preserved on GSC motility. GSCs were left either untreated or treated with Gint4.T, CtrlApt, CtrlApt-STAT3, or Gint4.T-STAT3 for 24 h, and their migration ability was monitored by Boyden chamber assays. As shown in Figure 4a–c and Figure S2a, the treatment with AsiC reduced cell migration by approximately 60–65%, further enhancing the ability of Gin4.T to alter cell mobility (30% reduction). No reduction was found upon treatment with control aptamer or conjugate. Next, we analysed the AsiC ability to interfere with the invading capability of GSCs. To this end, pre-treated GSCs were plated on Matrigel-coated filters and allowed to migrate. We found that a cell’s ability to migrate through the Matrigel in the presence of 10% FBS was significantly prevented in the presence of Gint4.T (30–40% reduction) and further inhibited by STAT3 AsiC, reaching about 65% inhibition as compared to the control aptamer or conjugate (Figure 4d–f and Figure S2b).

These data indicate that in the context of the AsiC, the Gint4.T aptamer and STAT3 siRNA synergize to hamper stem cell migration and invasion. 

### 2.4. The Gint4.T-STAT3 as Specific Inhibitors of Primary GSCs 

**Figure 5:**
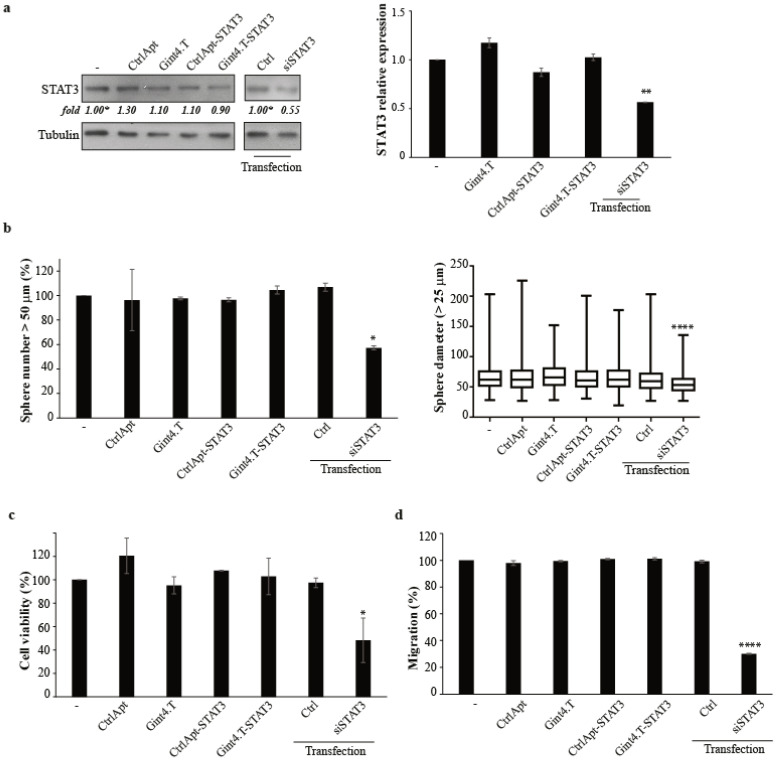
Gint4.T-STAT3 specificity. (**a**) GSC#144 (PDGFRβ^−^) were left untreated (−), treated with indicated aptamer or conjugates (400 nmol/L), or transfected with siSTAT3 (100 nmol/L) for 72 h, as indicated. Left panel: Cell lysates were immunoblotted with anti-STAT3 and anti-tubulin (used as a loading control) antibodies. Values below the blots indicate quantization relative to the control samples (labelled with asterisks) normalized on the loading control signals. Right panel: STAT3 mRNA levels were measured by RT-qPCR. Vertical bars indicate the mean ± SD (*n* = 2). (**b**) Sphere formation of GSC#144 (PDGFRβ^−^) left untreated (−), treated, or transfected, as indicated. Left panel: Spheres with a diameter > 50 μm were counted and expressed as the percentage relative to the untreated samples (−), set to 100%. Mean ± SD (*n* = 2) is reported. Right panel: Boxplot representation of diameter measures (spheres with a diameter > 25 μm). (**c**) Cell viability of GSC#144 (PDGFRβ^−^) following 72 h of indicated treatment (400 nmol/L) or transfection (100 nmol/L). Vertical bars: Mean ± SD (*n* = 2). (**d**) Cell migration of GSC#144 (PDGFRβ^−^) following 24 h of indicated treatment (400 nmol/L) or transfection (100 nmol/L). Vertical bars: Mean ± SD (*n* = 3). In (**c**,**d**), results are expressed as percentages with respect to the untreated cells. In (**a**–**d**), the statistics were obtained by one-way ANOVA: *, *p* < 0.05; **, *p* < 0.01; ****, *p* < 0.0001. Whole blots of Figure 5a are shown in Figure S8.

One key aspect of targeted delivery strategies is their ability to specifically act only on cells recognized by the targeting moiety. We thus attempt to demonstrate that the AsiC action on the GSCs was mediated by the aptamer recognition of the PDGFRβ. To this end, we treated a patient-derived GSC line (GSC #144) showing high levels of STAT3 but expressing low/undetectable levels of PDGFRβ (Figure S3). Notably, the AsiC treatment of GSC#144 did not change the STAT3 mRNA or protein levels (Figure 5a). Both the protein and mRNA were instead reduced when cells were transfected with the STAT3 siRNA, indicating that the AsiC-mediated silencing requires the presence of the PDGFRβ aptamer target. 

Further, we confirmed that the functional inhibitory actions of the AsiC were highly dependent on the presence of PDGFRβ. Thus, we analysed the ability of AsiC to affect tumour sphere formation in PDGFRβ-negative GSCs. As shown in Figure 5b, no change in GSC#144 sphere number and diameter was found upon AsiC treatment. On the contrary, both aspects were impaired when STAT3 silencing was forced by the transfection with STAT3 siRNA. Accordingly, the same results were obtained analysing cell viability by MTT assay (Figure 5c) or cell migration by using the Boyden chamber assay (Figure 5d and Figure S4). 

Taken together, these data indicate that the AsiC functionally acts in a receptor-dependent manner, allowing a specific targeting and inhibition of GSCs. 

### 2.5. The Gint4.T-STAT3 Acts in Combination with Anti-10b Aptamer Conjugate to Affect Tumour Sphere Formation

**Figure 6:**
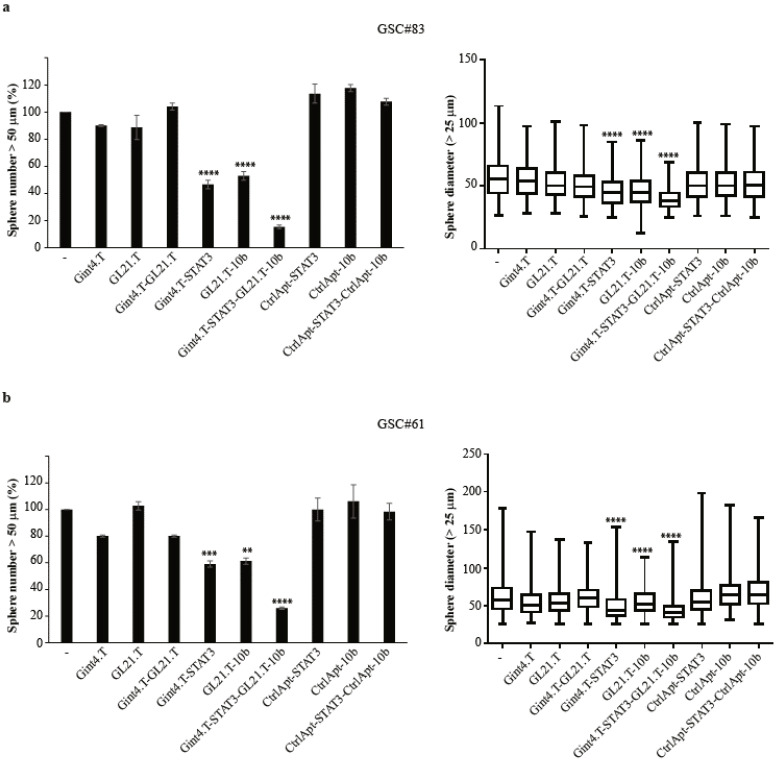
Gint4.T-STAT3 and GL21.T-10b combined effect on primary GSC tumour sphere formation. (**a**,**b**) Sphere formation of indicated primary GSC-derived tumour spheres (PDGFRβ^+^) left untreated (−) or treated with Gint4.T; Gint4.T-STAT3 or CtrlApt-STAT3; GL21.T-10b; or control aptamers—alone or in combination, as indicated. Left panels: Spheres with a diameter >50 μm were counted and expressed as the percentage relative to the untreated samples (−), set to 100%. Vertical bars depict the mean ± SD (*n* = 2). Right panels: Boxplot representation of the diameter measures (spheres with a diameter > 25 μm). Statistics of the conjugate treatments versus the untreated samples were obtained using one-way ANOVA: **, *p* < 0.01; ***, *p* < 0.001; ****, *p* < 0.0001.

In our previous report, we described the ability of an miR-10b antagonist (antimiR-10b) conjugated to Gint4.T or GL21.T aptamers to selectively target miR-10b and inhibit GSC stem-like phenotype and tumour sphere formation [24]. miR-10b is a biomarker that is highly expressed in GBM and GSCs, acting as an oncomiR to promote cancer stem cell propagation [8,22]. Although, STAT3 and miR-10b contribute to overlapping regulatory pathways, there is no evidence of direct expression regulation. We thus determined whether Gint4.T-STAT3 might synergize with the targeting of miR-10b. Since the efficiency of aptamer delivery is limited by the amount of target receptors on the cell surface [38], we took advantage of the GL21.T aptamer against the Axl receptor [24,35] to deliver the antimiR-10b to the GSCs (Figure S5a). The generated conjugate (GL21.T-10b) was used in combination with Gint4.T-STAT3 on GCS#83 and 61 that are positive for both PDGFRβ (Figure S1b) and Axl (Figure S5b) receptors. By analysing cell viability, we found that both Gint4.T-STAT3 and GL21-10b give a significant reduction that is not further enhanced upon their combination (Figure S5c,d). Conversely, conjugates efficiently synergize to affect tumour sphere formation (Figure 6). As shown, both conjugates interfere independently with sphere formation with similar efficacy, getting about a 50% reduction in the sphere number in the GSCs analysed. Notably, the combined treatment with the conjugates further decreased the number of spheres to approximately 20% (Figure 6, left panels) and size of spheres (Figure 6, right panels), thus strongly reducing GSC self-renewal potential. On the contrary, the treatments with control conjugates containing the control aptamer either alone or in combination are unable to affect sphere formation. 

These data underline the potential to combine Gint4.T-STAT3 with GL21.T-10b to drastically abrogate GSCs, enhancing the efficacy and the specificity of the treatment. 

## 3. Discussion

In the present study, we addressed the inhibition of GBM stem-like cells by an aptamer–siRNA molecule, combining the targeting of PDGFRβ and STAT3 gene silencing. We used a previously described nuclease resistant conjugate (Gint4.T-STAT3) [25] containing an aptamer (Gint4.T) that binds and inhibits the receptor tyrosine kinase PDGFRβ, [32], and a STAT3-specific siRNA. By using primary GSCs derived from patient with GBM tumours (WHO grade IV), we demonstrate the AsiC ability to alter self-renewal, viability, and migration.

The high intra-tumour heterogeneity of GBM is a serious impediment to the effectiveness of conventional anticancer chemo and radiotherapies, which preferentially targets bulk tumour cells while sparing the more resistant cell populations, such as GSCs [2,3,4,5,6]. Therefore, development of therapeutic strategies aimed at targeting the resistant population has become an urgent need to enhance the responsiveness to treatments. The deregulated activity of STAT3 in cancer cells as well as in GSCs makes it a very promising therapeutic target for the development of an effective strategy for a complete tumour eradication. 

On the other hand, in normal cells, STAT3 activity is tightly regulated by extracellular signals, enabling cells to respond to the microenvironment and maintaining a momentary active state [44]. The use of a targeting moiety for the selective silencing of STAT3 in cancer cells is thus imperative because it would permit to avoid the occurrence of severe side effects in normal tissues. A growing body of literature is demonstrating the selectivity of aptamer-mediated delivery that allows affecting only cells that express the aptamer target, thus sparing healthy tissues [45]. Accordingly, here we found that, at the difference of STAT3 siRNA transfection, the Gint4.T-STAT3 AsiC treatment is selective for GSCs that express high levels of the PDGFRβ aptamer target (Figure 5). We have recently described that the Gint4.T-STAT3 AsiC shows enhanced serum stability (up to 24 h in 80% serum) and is able to selectively drive the STAT3 siRNA to GBM differentiated cell lines [25]. In the present study, we addressed the efficacy of the Gint4.T-STAT3 treatment to target GSCs and suppress their self-renewing potential. We took advantage of different patient-derived primary human GSCs and found that upon AsiC treatment, the STAT3 gene is silenced (Figure 1), altering the stem-like phenotype and GSC propagation. Indeed, the treatment (1) hampered the formation of tumour spheres, decreasing the levels of Sox-2 and sustaining GFAP levels (Figure 2); (2) reduced the number of viable GSCs (Figure 3); and (3) inhibited GSC migration and invasion (Figure 4), enhancing the functional effect of the unconjugated Gint4.T aptamer [32]. 

We also found that Gint4.T-STAT3 synergizes with a chimera containing the anti-Axl aptamer GL21.T linked to the single chain antagonist of miR-10b (GL21.T-10b) [24]. Several miRNAs have been reported to be deregulated in GBM, governing different aspects of this tumour, including the maintenance and propagation of the GSCs [46]. Among others, miR-10b acts as an oncomiR and is required for GSC self-renewal and proliferation [9,23], and the targeted delivery of a miR-10b antagonist reduces GSC propagation [24]. Here we found that the combined treatment of Gint4.T-STAT3 with GL21.T-10b resulted in a synergistic and drastic inhibition of GSC self-renewal (Figure 6).

Numerous studies have shown that the STAT3 signalling pathway is required for the maintenance of the stem-like malignant glioma cells and that its inhibition by either chemical inhibitors, dominant-negative mutant protein, decoy oligodeoxynucleotides, or siRNAs can be a promising therapeutic strategy [47,48,49]. Our findings consolidate the therapeutic importance of STAT3 in GBM as a fundamental regulator of key tumour features. Most importantly, we provide a stable RNA-based molecule able to selectively inhibit STAT3, allowing impairing the maintenance of the GSC population. 

Despite many STAT3 inhibitors having been already developed, they still have a limited clinical use [50]. Peptide therapeutics are specific and potent but suffer from rapid degradation/instability and poor bioavailability (membrane permeability). Small molecule inhibitors, although being stable and able to cross the membrane efficiently, are less effective and specifically lead to unwanted side effects. In contrast, DNA and RNA oligonucleotides directed against STAT3 offer high specificity and efficacy, but membrane permeability and tissue-specific delivery remain limited. 

The multifunctional RNA bio-drug here described combines a cell-targeted inhibitory aptamer and a siRNA STAT3 antagonist offering the possibility to overcome the barriers to oligonucleotide therapies. Our results also show the possibility to design a combined approach targeting both STAT3 and miR-10b to affect the GSC population with high efficacy. 

In addition, the obtained data strongly support the idea of attacking GBM with innovative multiple therapies to enhance the success of the treatment; for example, associating STAT3 inhibitors with the drugs currently used in the clinic for GBM, such as temozolomide or bevacizumab [51]. 

Notably, recent evidence supports the ability of our targeting moieties to drive molecular carriers to the tumour site [24,52], thus sustaining the potential applicability of the described AsiC in GBM treatment, although its ability to successfully penetrate into intracranial GBM tumours remains to be determined. 

Collectively, our findings provide a proof-of-principle for the development of an AsiC-based therapeutic intervention with the potential to selectively target GSCs, potentially enhancing the current therapeutic treatment options. 

## 4. Materials and Methods 

### 4.1. Sequences 

Gint4.T, 5′-UGUCGUGGGGCAUCGAGUAAAUGCAAUUCGACA-3′;

Gint4.T stick, 

5′-UGUCGUGGGGCAUCGAGUAAAUGCAAUUCGACAXXXXGUACAUUCUAGAUAGCC-3′;

Aptamer used as control (CtrlApt),

5′-UUCGUACCGGGUAGGUUGGCUUGCACAUAGAACGUGUCA-3′;

Aptamer stick used in the control complexes,

5′-GCCGCUAGAACCUUCUAAGCGAAUACAUUACCGCXXXXGUACAUUCUAGAUAGCC-3′;

human STAT3 siRNA antisense (AS) strand stick,

5′-UUAGCCCAUGUGAUCUGACACCCUGAAGGCUAUCUAGAAUGUAC-3′;

human STAT3 siRNA sense strand (SS), 

5′-CAGGGUGUCAGAUCACAUGGGCUAA-3′

GL21.T, 5′-AUGAUCAAUCGCCUCAAUUCGACAGGAGGCUCAC-3′;

GL21.T stick,

5′-AUGAUCAAUCGCCUCAAUUCGACAGGAGGCUCACXXXXGUACAUUCUAGAUAGCC-3′;

antimiR-10b stick (indicated as anti-10b),

5′-CACAAAUUCGGUUCUACAGGGUAGGCUAUCUAGAAUGUAC-3′;

The STAT3 siRNA duplex sequences were previously reported [53]. All RNAs were produced by the DNA/RNA Synthesis Laboratory, Beckman Research Institute of City of the Hope or by Tebu-bio srl (Magenta, Milan, Italy). RNAs were modified with 2′-F-Pyrimidines. A stick-based approach [54,55,56] was adopted for complex generation. Stick sequences (underlined) within the complexes contained both 2′-F-Py and 2′-oxygen-methyl purines. A three-carbon linker ((CH2)3) spacer, indicated with the italic *X,* was included in the stick aptamers. Aptamers were refolded before each use by the following temperature cycle: 5 min at 85 °C, 3 min on ice, and 10 min at 37 °C. Complexes were prepared by two-step annealing: (1) the annealing of the STAT3 AS stick, with STAT3 SS done in an annealing buffer (20 mM 2-(4-(2- hydroxyethyl)piperazin-1-yl), ethane sulfonic acid (HEPES; pH 7.5), and 150 mM NaCl, 2 mM CaCl2) by incubating at 95 °C for 10 min, 55 °C for 10 min, and 37 °C for 20 min; and (2) to obtain the final complex, the AS–SS duplex was subsequently annealed with a stick aptamer incubating them at 37 °C for 30 min. For the generation of anti-10b complexes, the stick antimiR-10b, previously denatured at 95 °C for 10 min, was annealed to a refolded stick aptamer at 37 °C for 30 min. The complex formation was checked on a 12% non-denaturing polyacrylamide as the appearance of a shifted band. 

### 4.2. Cells and Transfection

The GSCs isolated from the tumour tissue of patients with GBM (WHO grade IV) were already published [38,39,40,41,57] and were provided by Dr Lucia Ricci-Vitiani. All the lines were grown in serum-free medium containing 20 ng/mL EGF and 10 ng/mL bFGF (Life technologies, Milan Italy), as described [37]. 

Before each treatment, tumour spheres were recovered by centrifugation (at 1000 rpm) and dissociated by using 0.25% trypsin. For treatments longer than 72 h, aptamers or conjugates were renewed three times a week at a 200 nmol/L concentration. 

For transfections, serum-free Opti-MEM and Lipofectamine 2000 reagent (Life technologies, Milan Italy) were used and the manufacturer’s protocol was followed with the annealed STAT3 siRNA (AS–SS duplex) at a 100 nmol/L concentration. 

### 4.3. Immunoblot Analysis and RT-qPCR Analysis

Dissociated tumour spheres (1.4 × 10^5^ cells/plate in 3.5-cm plates) were treated with 400 nmol/L aptamers or complexes or transfected, as indicated. JS buffer (50 mM Hepes (pH 7.5), 150 mM NaCl, 1% glycerol, 1% Triton X-100, 1.5 mM MgCl2, 5 mM EGTA, 1 mM Na3VO4, and protease inhibitors) was used for total cell lysates. Samples were prepared in sodium dodecyl sulfate/β-mercaptoethanol buffer and boiled before SDS-PAGE. The SDS-PAGE gels were blotted onto polyvinylidene difluoride membranes (Millipore, Billerica, MA, USA) by electrophoretic transfer. The following primary antibodies were used for immunoblots: anti-STAT3, anti-Sox-2, and anti-vinculin (Cell Signaling Technology Inc., Danvers, MA, USA); as well as anti-α-tubulin and anti-actin (used as a loading control) (Santa Cruz Biotechnology, CA, USA). Single bands at the expected molecular weight were considered (Figures S6–S9). Western blots were quantified by Image J NIH and band intensities (reported below the blots) were expressed as ratio normalized on the loading control signals. 

Gene mRNA levels were analysed by reverse transcription of 1 mg of total RNA with iScript cDNA Synthesis Kit followed by real-time PCR amplification with IQ-SYBR Green supermix (Bio- Rad, Hercules, CA, USA). The ΔΔCt method was used for relative mRNA quantization by applying the equation 2^−ΔΔCt^. Primers used were the following: STAT3: fw, 5′ ACCTGCAGCAATACCATTGAC 3′; rev, 5′ AAGGTGAGGGACTCAAACTGC 3′; GFAP: fw 5′ CTGCGGCTCGATCAACTCA 3′, rev. 5′ TCCAGCGACTCAATCTTCCTC 3′; Sox-2: fw 5′GCACATGAACGGCTGGAGCAAGC 3′, rev. 5′ TGCTGCGAGTAGGACATGCTGTAGG 3′; GAPDH (used as a housekeeping control): fw, 5′ CTTTGTCAAGCTCATTTCCTGG 3′; rev, 5′ TCTTCCTCTTGTGCTCTTGC 3′. 

### 4.4. Cell Count and Viability Assays

Tumour spheres were dissociated and 3 × 10^3^ cells/well (in 96-well plates) or 1.4 × 10^5^ cells/plate (in 3.5-cm plates) were seeded for cell viability or cell count assays, respectively. Cells were grown without treatment, treated with 400 nmol/L aptamers or complexes, or transfected, as indicated. Following 72 h, a CellTiter 96 Proliferation Assay (Promega, Madison, WI, USA) was used to measure cell viability or cells were counted after gentle pipetting. 

### 4.5. Transwell Migration and Invasion Assay

Dissociated tumour spheres were counted and 1.4 × 10^5^ cells/plate (in 3.5-cm plates) were seeded and grown with or without treatments for 24 h (400 nmol/L final concentration). Then, cells were recovered, washed, and 1 × 10^5^ cells/point were suspended in serum-free medium and seeded into a 24-well transwell (Corning Incorporate, Corning, NY, USA) upper chamber. The lower chambers were filled with 10% FBS (0.6 mL) used as an inducer of migration. Cells were allowed to migrate for an additional 24 h. To assess invasion, cells were plated in the upper chambers of a 24-well Transwell, previously coated with a 20% Matrigel matrix (BD Biosciences, San Jose, CA, USA). Cell staining with 0.1% crystal violet (in 25% methanol) was used to visualize the migrated or invaded cells. Crystal violet was eluted with 1% sodium dodecyl sulphate and the absorbance at 570 nm was read to quantify cell migration or invasion. 

### 4.6. Tumour Sphere Formation Assay

Dissociated tumour spheres were counted and 500 cells/well were seeded in a 96-well plate (in duplicate) and treated according to a previously published protocol [24]. Briefly, cells were untreated or treated with aptamers or complexes at 400 nmol/L concentrations. Treatments were renewed three times a week by the addition of 200 nmol/L aptamers or complexes. After 10 days of treatment, pictures of the spheres were acquired with a Leica Application Suite and the number of spheres (diameter > 25 μm) was counted. 

### 4.7. Statistical Analysis

For statistics, one-way ANOVA with multiple comparison was performed with GraphPad Prism. Student’s *t*-test was used to compare two groups. 

## 5. Conclusions

Here we demonstrated the ability of an aptamer–STAT3 RNA bio-drug to selectively target GSCs and inhibit their propagation. The molecule combines the inhibitory functions of the anti-PDGFRβ aptamer used as a targeting moiety and of the STAT3 siRNA cargo. 

Our results indicate that the STAT3 AsiC has the potential to selectively target the heterogonous complexity of GBM stem-like cells by interfering with multiple processes, including cell survival, migration, and stemness phenotype. Furthermore, the conjugate synergizes with the therapeutic targeting of miR-10b in inhibiting the elusive tumour-initiating GSC population. 

The study provides a proof-of-concept study that paves the way to the rational application of AsiC-based therapeutics for effective GBM therapy. 

## Supplementary Materials

The following are available online at https://www.mdpi.com/2072-6694/12/6/1434/s1, Figure S1: Gint4.T-STAT3 and primary GSCs, Figure S2: Gint4.T-STAT3 effect on GSC migration and invasion, Figure S3: PDGFRβ, STAT3 expression in primary GSC#144, Figure S4: Gint4.T-STAT3 effect on GSC#144 migration, Figure S5: Cell viability with Gint4.T-STAT3 and GL21.T-10b combination, Figure S6: Whole blots of Figure 1, Figure S7: Whole blots of Figure 2d; Figure S8: Whole blots of Figure 5a, Figure S9: Whole blots of supplementary figures.
