# Supplementary material for: Combined Targeting of Glioblastoma Stem-Like Cells by Neutralizing RNA-Bio-Drugs for STAT3
Source: Cancers (Basel). 2020 May 31;12(6):1434. doi: 10.3390/cancers12061434 (PMC7352497; doi:10.3390/cancers12061434)
Supplement: Supplementary file 1 [file cancers-12-01434-s001.pdf]

Supplementary Materials

Combined Targeting of Glioblastoma Stem-Like Cells by Neutralizing RNA-Bio-Drugs for STAT3

Carla Lucia Esposito, Silvia Nuzzo, Maria Luigia Ibba, Lucia Ricci-Vitiani, Roberto Pallini, Gerolama Condorelli, Silvia Catuogno and Vittorio de Franciscis

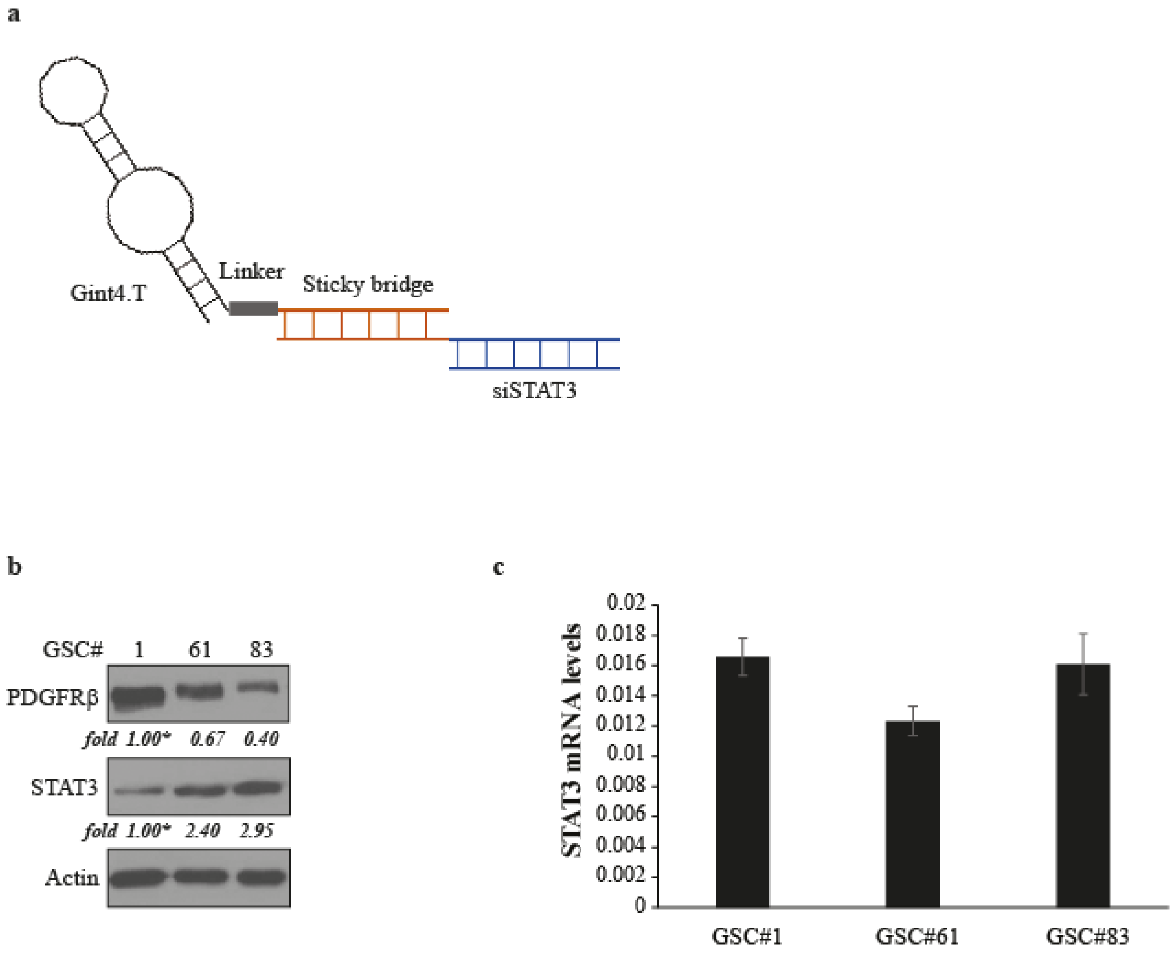

Figure S1. Gint4.T-STAT3 and primary GSCs. (**a**) scheme of Gint4.T-STAT3 conjugate. (**b**) Levels of PDGFRβ, STAT3 and actin (used as a loading control) were analysed by immunoblot in the indicated primary GSCs. Values below the blots indicate quantization ratio relative to GSC#1, labelled with asterisk normalized on the loading control signals. (**c**) Levels of STAT3 mRNA analysed by RT-qPCR in the indicated primary GSCs.

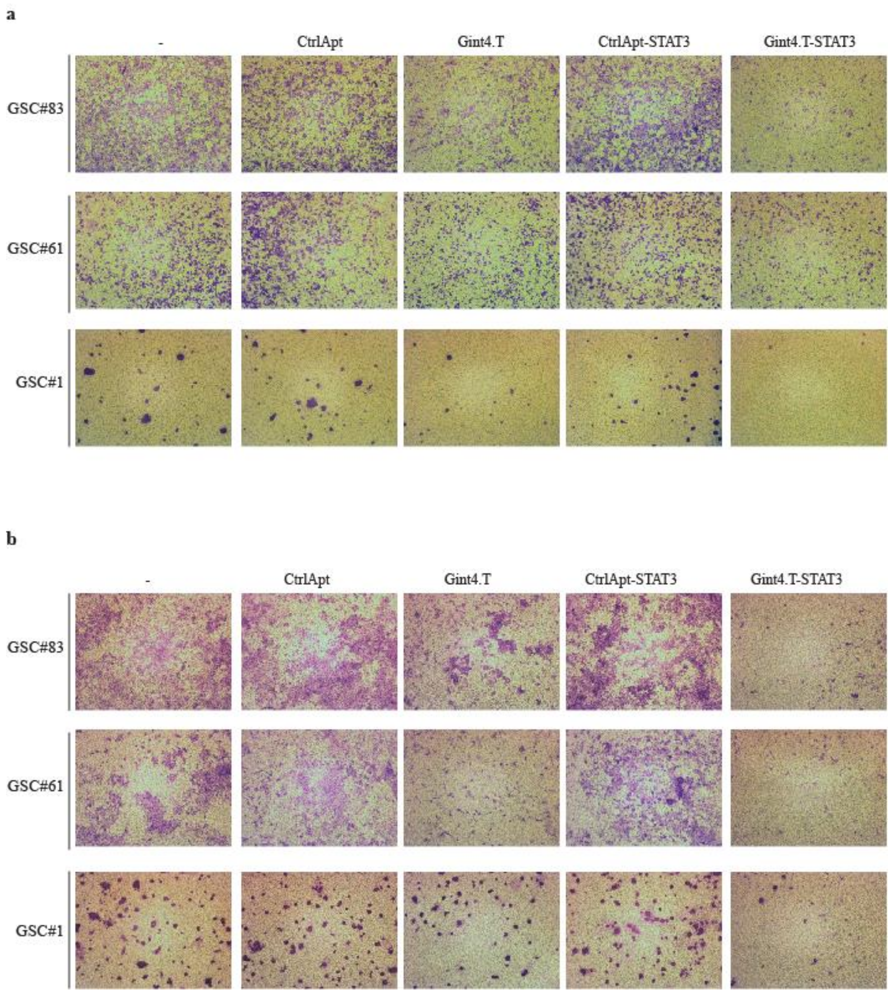

Figure S2. Gint4.T-STAT3 effect on GSC migration and invasion. Cell motility (**a**) or invasion (**b**) of indicated GSCs (PDGFRβ^{+}) left untreated or treated with indicated aptamers or conjugates (400 mol/L) for 24 hours was analysed. Representative micrographs are shown. Magnification, 10×.

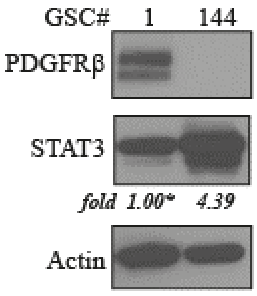

Figure S3. PDGFRβ, STAT3 expression in primary GSC#144. Levels of PDGFRβ, STAT3 and tubulin (used as a loading control) were analysed by immunoblot in the indicated primary GSCs. Values below the blots indicate quantization relative to GSC#1, labelled with asterisk normalized on the loading control signals.

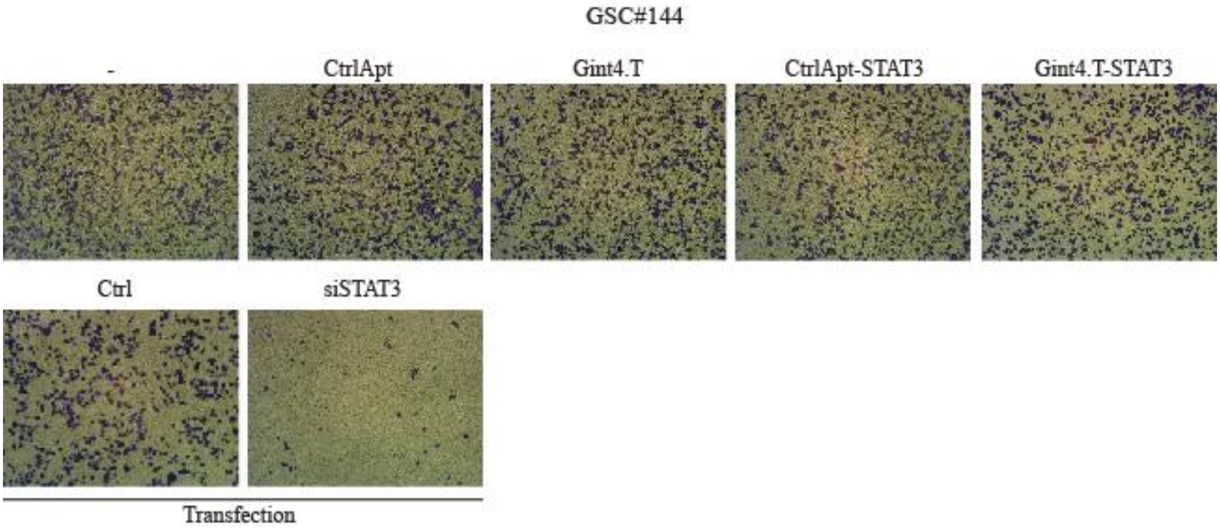

Figure S4. Gint4.T-STAT3 effect on GSC#144 migration. Cell motility of GSC#144 (PDGFRβ^{-}) left untreated (−), treated with indicated aptamers or conjugates (400 mol/L) or transfected with siSTAT3. Representative micrographs are shown. Magnification, 10×.

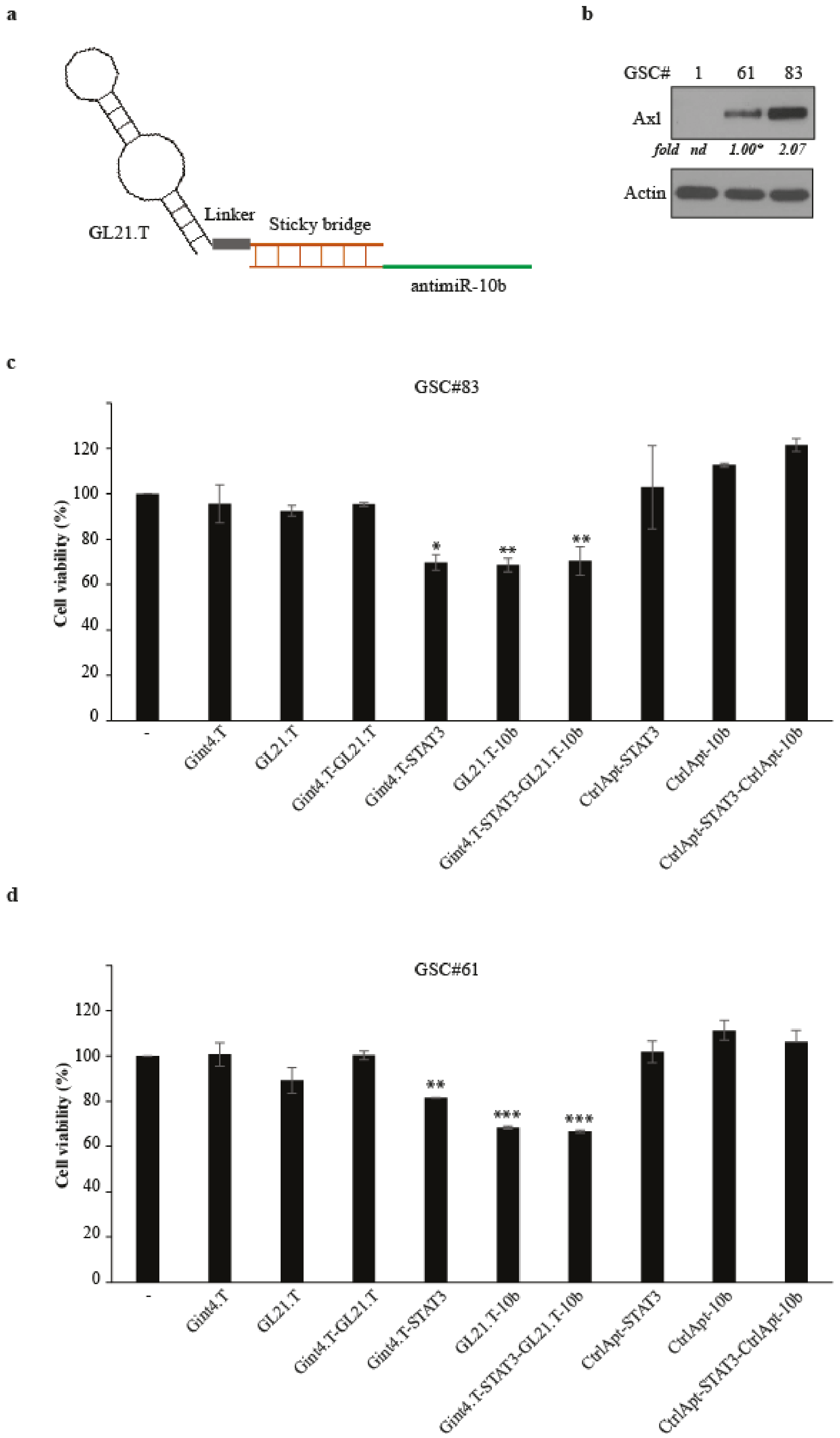

Figure S5. Cell viability with Gint4.T-STAT3 and GL21.T-10b combination. (**a**) Scheme of GL21.T-10b conjugate. (**b**) Levels of Axl and actin (used as a loading control) were analysed by immunoblot in the indicated primary GSCs. Values below the blots indicate band intensity ratio normalized on the loading control signals. (**c**,**d**) GSC#83 (c) or 61 (d), (PDGFRβ$^+$, Axl$^+$), were left untreated (−) or treated with indicated aptamer or conjugates (400 mol/L) for 72 hours alone or in combination. Cell viability was measured and expressed as per cent of viable cells with respect to untreated cells. Statistics versus untreated samples by Student's *t* test: *, $p < 0.05$; ** $p < 0.01$; ***, $p < 0.001$. Vertical bars depict mean ± SD.

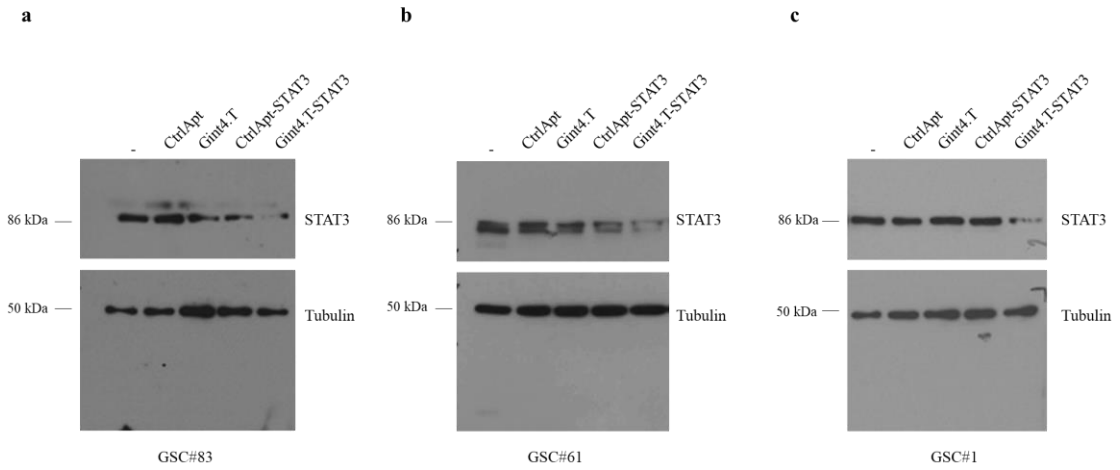

Figure S6. Whole blots of Figure 1. (**a**,**c**) Whole blots of Figure 1 a, b and c (left panels), respectively. Filters were probed for: STAT3 (upper parts) or tubulin (lower parts). .

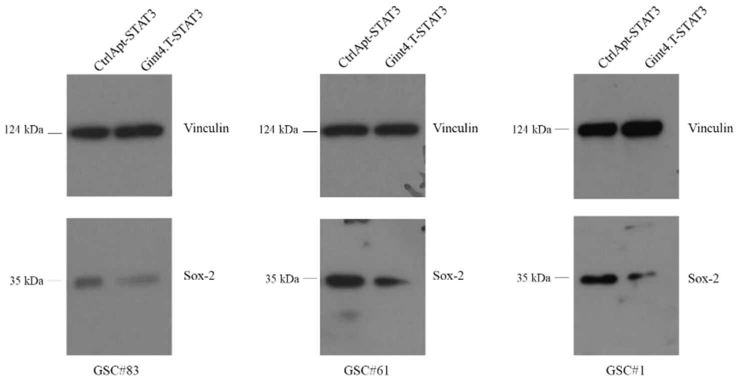

Figure S7. Whole blots of Figure 2d. Whole blots of Figure 2 d (right panels). Filters were probed for Sox-2 and vinculin antibodies.

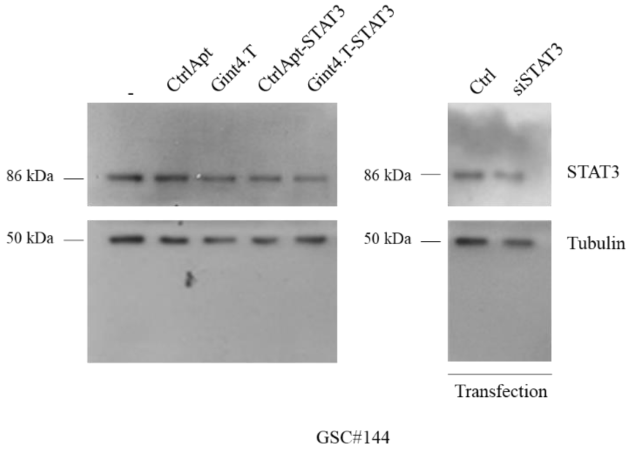

Figure S8. Whole blots of Figure 5a. Filters were probed for STAT3 (upper parts) or tubulin (lower parts).

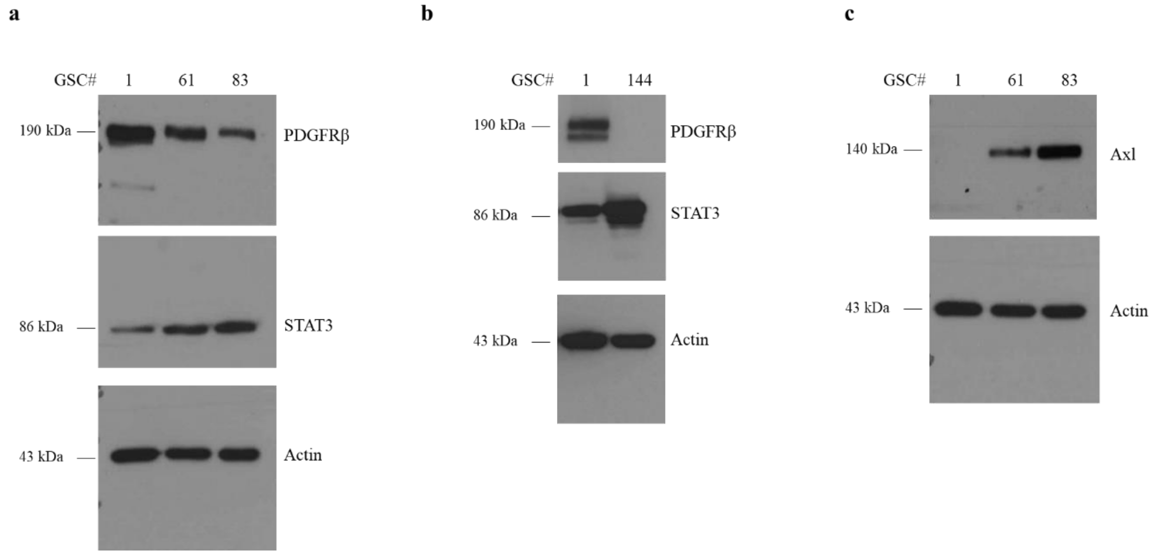

Figure S9. Whole blots of supplemenatry figures. (a) Whole blots of Figure S1a. The upper part was probed first with PDGFRβ and then with STAT3, the lower part was probed with Actin. (b,c) whole blots Figure S3 or Figure S5b, respectively.

© 2020 by the authors. Licensee MDPI, Basel, Switzerland. This article is an open access article distributed under the terms and conditions of the Creative Commons Attribution (CC BY) license (http://creativecommons.org/licenses/by/4.0/).
